# COVID-19 Pandemic as a Traumatic Event and Its Associations with Fear and Mental Health: A Cognitive-Activation Approach

**DOI:** 10.3390/ijerph18147422

**Published:** 2021-07-12

**Authors:** Martin Sanchez-Gomez, Gabriele Giorgi, Georgia Libera Finstad, Flavio Urbini, Giulia Foti, Nicola Mucci, Salvatore Zaffina, José M. León-Perez

**Affiliations:** 1 Department of Evolutionary, Educational, Social Psychology and Methodology, Universitat Jaume I, 12071 Castellón de la Plana, Spain; 2 Department of Human Science, European University of Rome, 00163 Rome, Italy; gabriele.giorgi@unier.it (G.G.); flavio.urbini@unier.it (F.U.); 3 Business@Health Laboratory, European University of Rome, 00163 Rome, Italy; g.liberafinstad@gmail.com (G.L.F.); giuliafoti.98@gmail.com (G.F.); 4 Department of Experimental and Clinical Medicine, University of Florence, Largo Piero Palagi 1, 50139 Florence, Italy; nicola.mucci@unifi.it; 5 Occupational Health Unit, Medical Direction, Bambino Gesù Children’s Hospital IRCCS, 00165 Rome, Italy; salvatore.zaffina@opbg.net; 6 Department of Social Psychology, Universidad de Sevilla, 41004 Sevilla, Spain

**Keywords:** COVID-19, mental health, PTSD, pattern, intrusion, hyperarousal, avoidance

## Abstract

The COVID-19 global pandemic still represents a major threat with detrimental health consequences. Analyzing the psychological outcomes, COVID-19 could be interpreted as a collective traumatic event that can generate symptoms related to post-traumatic stress disorder (PTSD). Considering this, the purpose of this paper is twofold: first, to investigate the relationship between intrusive thoughts and fear related to the COVID-19 pandemic and between intrusive thoughts and mental health; second, to test the mediating role of hyperarousal and avoidance in these two relationships. In order to reach these aims, the present study investigated these relationships and tested a mediation model in two cross-sectional studies in Italy. Altogether, 627 individuals and 495 workers completed an online survey for study 1 and study 2, respectively. Mediation analyses were performed via the SPSS macro PROCESS; the significance of total, direct, and indirect effect was tested via bootstrapping. The results showed that within the PTSD framework, hyperarousal compared with avoidance mediated the relationship between intrusion and the analyzed outcomes. In conclusion, the present study provided empirical evidence for the influence of hyperarousal on individual consequences such as fear of COVID-19 and mental health. Research, as well as theoretical and practical implications, are discussed.

## 1. Introduction

Since the World Health Organization (WHO) declared a state of international health emergency due to the Coronavirus outbreak, COVID-19 has led to an unexpected evolution of contagion and a scenario of death and isolation. Many countries have adopted lockdown measures and have radically changed their lifestyles by switching to protective devices and social distancing. Several studies carried out during the pandemic testify to the significant impact COVID-19 pandemic has had on the mental health of individuals, causing stress, anxiety, depressive symptoms, insomnia, denial, anger, and fear [1]. The quarantine status led to negative psychological consequences like health anxiety, financial worry, and loneliness [2]. In addition, “headline stress disorder” can be observed during this pandemic. This disorder is characterized by high emotional response (such as stress and anxiety) to the endless media reports that may cause physical symptoms including palpitation and insomnia [3]. Therefore, COVID-19 pandemic can be interpreted, to all intents and purposes, as a collective traumatic event that can generate posttraumatic symptoms [4]. From this perspective, research and clinical practice showed that trauma per se is a powerful risk factor for mental disorders being posttraumatic stress disorder (PTSD) characteristic of the most common ones [5,6]. PTSD can be defined as a disorder that may occur in people who have experienced or witnessed a traumatic event such as, for example, a natural disaster [5]. In this regard, recent scientific evidence has highlighted the existence of a relationship between the COVID-19 pandemic and increased levels of PTSD [7,8]. According to the International Classification of Diseases (ICD-11) [9], PTSD is composed of three core symptoms: ‘Intrusions’ or intrusive thoughts such as repeated and involuntary memories or concerns about the traumatic event that interrupt a flow of thought (also distressing dreams or flashbacks of the traumatic event, which can be so vivid that people feel they are re-living the traumatic experience); ‘Avoidance’ or purposefully avoiding people, places, activities, objects, and situations that may trigger distressing memories related to the traumatic event; and ‘Hyperarousal’ or excessive vigilance that occurs with exaggerated startle response, difficulty in concentrating or remembering. These symptoms were reported in the first studies conducted on the COVID-19 emergency in China and Italy [7,10,11].

Within the literature, intrusion seems to be the most frequent symptom followed by hyperarousal and avoidance [12]. More specifically, intrusive thoughts compared to other elements are recognized as a hallmark and troublesome features of PTSD [13]. However, as Bridgland and colleagues noticed, most theoretical models do not account for potential threats looming in the future as the causes of PTSD [8]. In response, we integrate a three-dimensional approach to study PTSD (e.g., intrusion, hyperarousal, and avoidance) with the Cognitive-Activation Theory of Stress (CATS) to address the relationship between the dimensions of PTSD (i.e., intrusion, hyperarousal, and avoidance) and the COVID-19 pandemic-related fear and mental health in different Italian samples [14,15,16,17,18].

The emphasis on identifying the optimal structural model of PTSD has at least two main practical implications: a direct implication in diagnostic procedure and the assessment of comorbidity with other psychopathologies [19,20]. 

### 1.1. COVID-19 Pandemic Trauma and Fear Feelings

COVID-19 has led to a drastic scenario of infections and serious consequences for the health of individuals. Furthermore, the pandemic spreads a general fear that dramatically affects people’s lives. Specifically, anxiety is due to two reasons; fear of infection and the symptomatic consequences that may result from it [21,22].

Recent research conducted in Pakistan investigated the presence, intensity, and dynamics of fear of the coronavirus among general population [23]. Participants were given an online questionnaire, which included information on socio-personal data and closed-and-open-ended questions regarding coronavirus fear. Respondents who had a high level of fear were asked to describe its nature. The results showed that the level of fear was higher for women. Furthermore, nine main themes were extrapolated from their responses: ‘Corona fear’ (fear of the disease itself, fear of not receiving treatment, fear of falling ill, fear of spreading the disease to family members, contagiousness, timelessness of the disease, rapid spread, and the burden of caring for the family if infected), loss (loss of loved ones and loss of job), fear of isolation (fear of living away from one’s family and staying at home for a long time), fear associated with religion, fear of death (fear of dying, fear of the death of others, fear of dying before reaching one’s goals and having a horrible death), the consequences of COVID-19 in terms of blocking the future, the underdevelopment of the country, fear of psychological consequences (sense of powerlessness, sense of uncertainty, fear of being wrong and stress/depression, anxiety about increased mortality, dependence and inactivity) and finally, empathy (for the poorest people and concern for the global spread of the disease).

Furthermore, fear of COVID-19 has consequences for both physical and psychological health and a greater impact on the most vulnerable population. This is also confirmed by the results of a study conducted in the United States that investigated the prevalence of fear and its consequences, describing the variation among the most socially vulnerable sub-populations [24]. The study revealed a population that is worried, afraid, and uncertain about the pandemic situation and its consequences for the individual, the family, and the community as a whole. Fear of contagion pervades multiple aspects of life to the point of being a pervasive thought that has also led the most vulnerable people to commit suicide. In particular, a study carried out in India examined 69 cases of suicide and analyzed related media information [25]. It has been hypothesized that most of the suicides were caused by the fear of contracting the infection, although after the autopsy most of the subjects tested negative for COVID-19. Furthermore, the prevalence of men in suicide cases was higher regardless of age group (19–95 years).

In this sense, we follow a cognitive appraisal model of PTSD. These models suggest that cognitive factors, particularly appraisals of ongoing threat, are crucial to understand trauma response. For example, Horowitz’s (1982) model of PTSD, considers intrusion as the primary factor in the onset of post-traumatic symptoms that may precede hyperarousal and avoidance responses. In turn, this activation (i.e., hyperarousal) and avoidance may increase negative feelings such as fear of the COVID-19 pandemic. In this aspect, according to CATS theory, the COVID-19 pandemic can be interpreted as a traumatic experience itself associated with PTSD symptoms [7,8]. Furthermore, this trauma elicits intrusive thoughts, which can be considered as a threatening stressor (i.e., cognitive activation) that sustains a physiological activation (i.e., hyperarousal) that leads the individual to cope with the stressor (in this case, to avoid the stressor or traumatic event) [26]. Then, as the exposure to the COVID-19 pandemic continues, the cognitive-activation persists (and probably repeatedly activates the HPA axis) with its potentially negative consequences in terms of both increased negative feelings such as anger, irritability, or fear, and diminished mental health.

**Figure 1:**
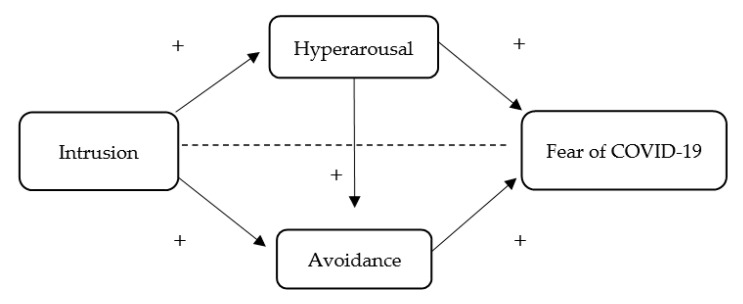
Sequential mediation model proposed to test the associations between intrusive thoughts, hyperarousal, avoidance, and fear of COVID-19 (Study 1). +: positive relationship.

Therefore, taking as a baseline these previous findings, the first aim (Study 1) was to investigate the relationship between the dimensions of PTSD (i.e., intrusion, hyperarousal, and avoidance) and the COVID-19 pandemic-related fear among general population. In particular, we investigated whether the relationship between intrusion and fear of COVID-19 is mediated by hyperarousal and avoidance. In other words, we hypothesize that the causal relationship between intrusion and fear of COVID-19 will be mediated by hyperarousal and avoidance. The proposed multiple sequential mediation model can be seen in Figure 1.

### 1.2. COVID-19 Pandemic Trauma and Mental Health

The results of the referred literature highlight the extent to which the COVID-19 pandemic may have a significant impact on the psychological health of individuals. A recent research conducted in Hubei province attempted to extrapolate the main symptoms of PTSD as a result of the pandemic. In the network of COVID-19 pandemic-related PTSD symptoms, results showed strong connections between avoidance of thoughts and avoidance of reminders, between hypervigilance and exaggerated alarm response, between intrusive thoughts and nightmares, between flashbacks and hyperresponsiveness to emotional signals, and between detachment and limited affection. Furthermore, the study suggested that the main symptom was self-destructive/reckless behavior, which was positively correlated with the presence of depression and loss of interest [27].

Furthermore, previous studies have shown an association between PTSD symptoms related to the COVID-19 pandemic and several measures of mental health, including anxiety, depression, and psychological functioning [8]. For example, a meta-analysis examined the effect of 62 studies addressing the impact of PTSD symptoms on general health and concluded that people with high levels of PTSD symptoms also reported poorer health outcomes [28]. Similarly, a study of 168 returning veterans found that PTSD symptoms have a unique contribution to mental health (6%) when controlling for the effect of several predictors such as severity of trauma exposure, physical injury, or substance abuse [29].

**Figure 2:**
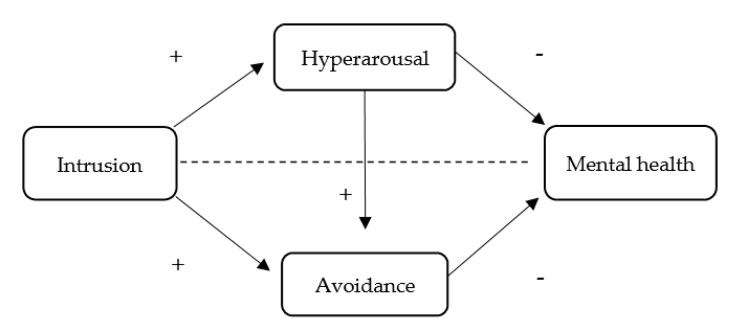
Sequential mediation model proposed to test the associations between intrusive thoughts, hyperarousal, avoidance, and mental health (Study 2). +: positive relationship; −: negative relationship.

Therefore, following the same rationale as in the previous section, we assume that the COVID-19 pandemic is a traumatic event that leads to experiencing PTSD symptoms, which, in turn, are associated to poorer mental health. As mentioned above, based on CATS theory and cognitive appraisal models of PTSD, cognitive mechanisms (i.e., intrusion) produce a physiological activation (hyperarousal) that prompts the coping response (avoidance in this case, as the event cannot be controlled or solved); however, as this cognition-activation persists and the coping strategies are maladaptive or unsuccessful, individuals’ mental health is negatively affected. Thus, the second aim (Study 2) was to investigate the relationship between the PTSD dimensions (i.e., intrusion, hyperarousal, and avoidance) and mental health in a sample of Italian workers. In this case, we hypothesized that the relationship between intrusion and mental health will be mediated by hyperarousal and avoidance. This mediation model is illustrated in Figure 2. 

## 2. Materials and Methods

### 2.1. Sample and Design

In Study 1, we followed a cross-sectional design. The study was developed during the second half of 2020 and early 2021, in parallel with the COVID-19 pandemic. Study 1 sample consisted of 627 subjects. Table 1 shows the socio-demographic characteristics of the participants. 

Similarly, Study 2 followed a cross-sectional design and was developed during the second half of 2020 and the beginning of 2021. The sample of the second study consisted of 495 workers selected from several Italian companies. Table 2 shows the socio-demographic characteristics of these participants.

#### 2.1.1. PTSD Generated by COVID-19

The Impact of Event Scale in its six-item version was used to assess the stress generated by the COVID-19 pandemic (IES-6) [30]. For this purpose, the items were translated into Italian. The respondents were instructed to answer the questionnaire considering the Coronavirus pandemic as the potentially stressful event. This questionnaire is an abbreviated version of the original IES-R, a 22-item screening test to assess posttraumatic stress disorder (PTSD) [31]. The items are related to feelings of distress experienced over the last 7 days, expressly following a specific traumatic situation, which, in our case, is the COVID-19 pandemic. Following a five-point Likert scale, the IES-6 includes two items for each of the dimensions of posttraumatic stress: intrusion (e.g., “Since the beginning of the COVID-19 emergency, I thought about it when I didn’t mean to”), avoidance (e.g., “Since the beginning of the COVID-19 emergency, I was aware that I still had a lot of feelings about it, but I didn’t deal with them”) and hyperarousal (e.g., “Since the beginning of the COVID-19 emergency, I had trouble concentrating”). The internal reliability of the variables was adequate (Study 1: Intrusion = 0.85, Hyperarousal = 0.82 and Avoidance = 0.66; Study 2: Intrusion = 0.86, Hyperarousal = 0.73 and Avoidance = 0.66). This scale was used in both Study 1 and Study 2.

#### 2.1.2. Fear of COVID-19

We measured the extent to which the pandemic constituted a threat to people with an eight-item scale developed for this purpose (e.g., I am afraid of contracting the virus). The scale uses a five-point Likert scale ranging from 1 (strongly disagree) to 5 (strongly agree). A total score is calculated by obtaining the sum of all eight items. The higher the score, the greater the fear of COVID-19. This questionnaire demonstrated a stable unidimensional structure and showed adequate psychometric properties (see Table 3). Moreover, its internal consistency was high (α = 0.90). This scale was used only in Study 1.

#### 2.1.3. Mental Health

The Italian version of the General Health Questionnaire (GHQ-12) was used in order to assess the symptoms of mental distress [32]. This is a self-administered questionnaire adapted from the original developed by Goldberg and Williams to assess non-specific psychiatric disorders [33]. Following a four-point Likert-scale, participants answered 12 items based on their current experience of mental distress (e.g., I was unable to enjoy daily activities). It is important to note that in this study, unlike the original questionnaire, the scoring was performed in the opposite direction. Thus, the higher the score, the greater the mental health. Cronbach’s alpha values indicate a good level of reliability (α = 0.86). This scale was used only in Study 2.

### 2.2. Procedure

Following similar previous studies, participants in Study 1 were recruited through psychology graduates and PhD students who had experience in psychological assessment. The procedure was performed based on the recommendations offered by Wheeler et al. to apply this type of sampling technique [34]. The test battery was developed using the Google Forms platform and was emailed using a research lab database of 2702 people. 627 subjects completed the full form (response rate = 23.2%). The format included a first page in which it was mandatory to demonstrate a minimum age of 18 and in which the voluntary and confidential nature of the collaboration was clarified. All the participants accepted the conditions of this research. The whole process was conducted in accordance with the Declaration of Helsinki. Given the observational nature of the study along with the absence of any involvement of therapeutic medication, no formal approval of the Institutional Review Board of the local Ethics Committee was required. Furthermore, the American Psychological Association’s (APA) Ethical Principles of Psychologists and Code of Conduct were followed.

In Study 2, participants were recruited from several Italian companies following a convenience sampling procedure. Firstly, the researchers of this study contacted several companies from various sectors and informed Human Resources (HR) managers of the opportunity to participate. Once accepted, an email invitation was sent to 786 people, 495 of whom responded (response rate = 62.9%). All participants agreed to participate voluntarily in the research and stated that they were at least 18 years old. The questionnaires were administered through the Google Forms platform. The whole process was performed following the APA Ethical Principles and Code of Conduct and in accordance with the Declaration of Helsinki. Given the observational nature of the study, and in the absence of any involvement of therapeutic medication, no formal approval of the Institutional Review Board of the local Ethics Committee was required.

### 2.3. Data Analysis

The statistics software IBM SPSS^®^ (v. 26, package for Windows, SPSS Inc., Chicago, IL, USA) was used to analyze the data. Initially, the distribution of the variables was analyzed using the Kolmogorov–Smirnov test to check for normality. After establishing the normality of the distribution, the descriptive statistics, including the mean and the standard deviation and Pearson correlations between Intrusion (independent variable), hyperarousal (first mediator), avoidance (second mediator), and fear of COVID-19 (dependent variable of Study 1)/mental health (dependent variable of Study 2) were obtained. Secondly, reliability analyses were performed for the study variables. Furthermore, the SPSS macro PROCESS 3.3 (Andrew F. Hayes, AB, Canada) was used to test the proposed associations regarding the mediation models (Figure 1 and Figure 2) [35]. Then, a standard procedure was followed using a 10,000 bootstrap sample, which produced 95% bias-corrected confidence intervals. A path is statistically significant if the associated 95% confidence interval (CI; bias corrected) does not include zero. The level of significance was set at *p* ≤ 0.05.

## 3. Results

### 3.1. Descriptive Analyses

Descriptive statistics (i.e., means, standard deviations) and bivariate correlations between the study variables regarding Study 1 and Study are described in Table 4 and Table 5, respectively. As expected, intrusion correlated positively with hyperarousal (r = 0.65 in Study 1; r = 0.63 in Study 2), avoidance (r = 0.53 in Study 1; r = 0.52 in Study 2), and fear of COVID-19 (r = 0.42 in Study 1). Also, Intrusion correlated negatively with mental health (r = −0.25 in Study 2). Furthermore, hyperarousal (r = 0.41) and avoidance (r = 0.36) were positively correlated to fear of COVID-19 (Study 1); whereas hyperarousal (r = −0.49) and avoidance (r = −0.43) were negatively correlated to mental health (Study 2).

### 3.2. Multiple Mediation Analyses

**Figure 3:**
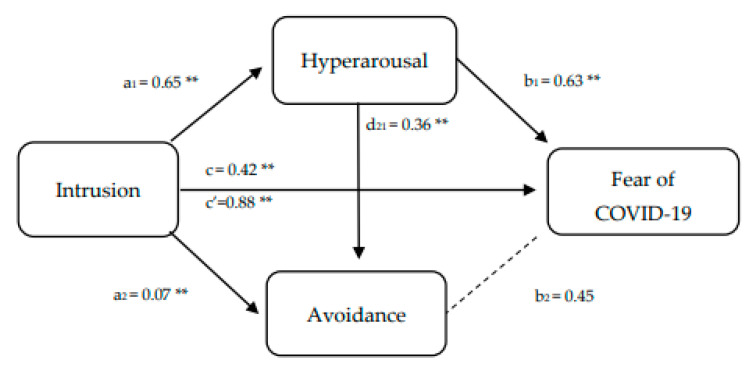
Sequential mediation model proposed to test the associations between intrusive thoughts, hyperarousal, avoidance, and fear of COVID-19. ** *p* < 0.01.

The first multiple mediation analysis was performed to test the associations between intrusive thoughts, hyperarousal, avoidance, and fear of COVID-19. As can be seen in Figure 3, intrusion was positively and significantly related to hyperarousal (a_1_ = 0.65; *p* < 0.01), and avoidance (a_2_ = 0.07; *p* < 0.01). The relationship between intrusion and fear of COVID-19 (c = 0.42; *p* < 0.01) was partially mediated by hyperarousal. Meanwhile, the direct effect kept its significance (c’ = 0.88; *p* < 0.01). Once the multiple mediation pathways were tested, only path 1 (a_1_b_1_ = intrusion–hyperarousal–fear of COVID-19) was significant (B = 0.41; SE = 0.13; 95% CI = 0.14; 0.67). Thus, higher intrusion activity resulted in higher hyperarousal, which increased the fear of COVID-19. 

**Figure 4:**
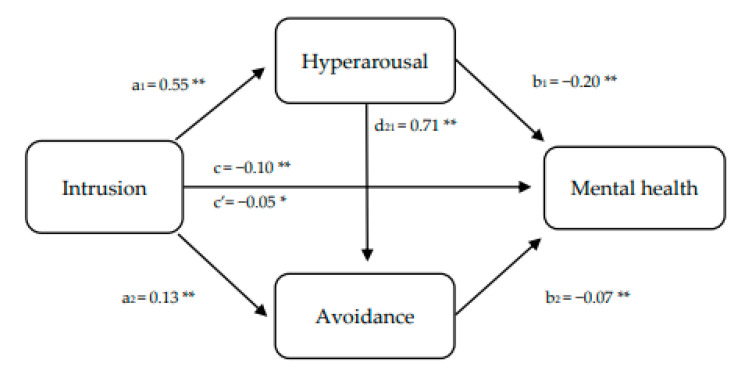
Sequential mediation model proposed to test the associations between intrusive thoughts, hyperarousal, avoidance, and mental health. * *p* < 0.05. ** *p* < 0.01.

The second multiple mediation analysis was performed to test the associations between intrusive thoughts, hyperarousal, avoidance, and mental health. As can be seen in Figure 4, intrusion was positively and significantly related to hyperarousal (a_1_ = 0.55; *p* < 0.01), and avoidance (a_2_ = 0.13; *p* < 0.01). The relationship between intrusion and mental health (c = −0.10; *p* = 0.02) was partially mediated by hyperarousal and the direct effect continued to be significant (c’ = −0.05; *p* < 0.01). Once the multiple mediation pathways were tested, three significant paths were found: path 1 (a_1_b_1_ = intrusion-hyperarousal-mental health; B = −0.11; SE = 0.02; 95% CI = 0.07; 0.15), path 2 (a_1_d_21_b_2_ = intrusion-hyperarousal-avoidance-mental health; B = −0.07; SE = 0.02; 95% CI = 0.02; 0.12), and path 3 (a_2_b_2_ = intrusion-avoidance-mental health; B = −0.02; SE = 0.01; 95% CI = 0.01; 0.04). After examining the pairwise contrasts of indirect effects, the first path was found to be the most important. Thus, greater intrusion activity resulted in higher hyperarousal, which reduced perceived mental health. 

## 4. Discussion

This study aimed to further-develop the understanding of the consequences of COVID-19 pandemic within the PTSD framework. Specifically, hyperarousal and avoidance have been suggested as possible underlying mechanisms between intrusion, considered as the primary factor in the onset of post-traumatic symptoms of PTSD, and two different outcomes, that is, fear of COVID-19 and mental health. Nowadays, PTSD is widely recognized as one of the most probable psychosocial consequences of the COVID-19 pandemic (e.g., [11]). Although interest on the psychological impact of COVID-19 pandemic is increasing (e.g., [36]) existing research has not fully considered yet the role of PTSD and its consequences on mental health and COVID-19 related issues. In the present research, two studies were designed to address this gap by developing and testing two independent models in which two dimensions of PTSD, hyperarousal and avoidance, mediated the effects of intrusion on two individual outcomes. Study 1 examined the association of intrusion with a type of individual reaction closely related to COVID-19: fear of COVID-19. Study 2 investigated the relationship between intrusion and mental health. Both models are drawn on the theoretical model of Horowitz, which considers intrusive thoughts as a factor that may precede avoidance and hyperarousal [37]. 

The current findings provided further evidence for this approach. First, the results supported the relationship between the variables. In this regard, in Study 1 intrusion correlated positively with hyperarousal, avoidance, and fear of COVID-19. Furthermore, hyperarousal and avoidance were negatively related to fear of COVID-19. In Study 2, intrusion correlated positively with hyperarousal and avoidance and negatively with mental health. Similarly to Study 1, hyperarousal and avoidance were negatively correlated with mental health. With respect to path model in study 1, intrusion had a positive and significant direct effect on fear of COVID-19; furthermore, the indirect effect of intrusion on fear of COVID-19 via two mediators (hyperarousal and avoidance) was positive and significant only for hyperarousal. Regarding the path model in Study 2, intrusion had a positive and significant direct effect on mental health. Furthermore, the indirect effect of intrusion on fear of COVID-19 via two mediators (hyperarousal and avoidance) was negative and significant only in the case of hyperarousal. Therefore, hyperarousal mediated the relationship between intrusion and two individual outcomes, emphasizing the key role that hyperarousal plays in the domain of PTSD. Despite the fact that intrusive symptoms can generate higher levels of avoidance, this study highlighted the prevalence of hyperarousal over it [38]. 

This result supports the idea that COVID-19 pandemic is a pervasive and specific mass traumatic event that people fundamentally perceive by shifting to a position of hypervigilance rather than avoidance. Such findings are coherent with the CATS theory, which proposes that cognition leads to physiological activation [18]. Practically speaking, the contingent situation makes people constantly think about the traumatic event of the COVID-19 pandemic and this leads to feel nervous and alarmed, having excessive vigilance. Moreover, as they cannot avoid the traumatic event and get rid of the stressor, they experience negative emotions and fear subsequently affecting their mental health in a harmful manner.

The present study highlighted the key role of hyperarousal in the relationship between intrusive thoughts and fear of COVID-19 and intrusive thoughts and mental health, showing its strategic role for a better understanding of psychological health promotion strategies. In summary, our findings broaden research findings on COVID-19 and PTSD, as they can be used and applied to better explain the individual consequences of the COVID-19 pandemic. 

### 4.1. Limitation and Future Directions

Despite the precautions, the present work has several limitations that need to be considered in the future. First, despite having stated the theoretical foundations that support our proposals, the current research followed a cross-sectional design, which does not allow for causal inference about the relationships among the study variables. Future research should employ longitudinal designs to replicate these findings and shed light on how PTSD could lead to different individual outcomes such as fear of COVID-19 and mental health consequences. Furthermore, future studies are needed to support the link between the three fundamental factors of PTSD. In this regard, we are aware that the model tested via a cross-sectional study is not the best way to examine a mediation effects [39]. However, we are supported by both theoretical and empirical evidence so that we can be a little more confident that the direction may follow the proposed path [13,37]. Future research needs to address the potential bidirectional associations between PTSD symptoms and negative emotions and possible negative spirals in which fear of the COVID-19 pandemic can reinforce intrusive thoughts and maintain an elevated physiological activation or, in contrast, potential adaptation to the traumatic event that may help people cope with the situation [40,41].

A second weakness is related to the non-probabilistic sampling technique. In both studies, the samples were recruited following a convenience sampling procedure, which may have undermined the generalization of our results. However, as described above, both samples were composed of a heterogeneous group of people, covering different age groups with a balanced gender distribution. Moreover, the recommendations offered by Wheeler et al. were followed [34]. This perspective supports this method of data acquisition as it has shown good levels of validity and reliability and is usually employed in organizational psychology. Furthermore, the COVID-19 pandemic forced this type of data collection inasmuch as traditional paper and pencil questionnaires were very difficult to administer. Future research should test the generalizability of these results in larger representative samples. There is another limitation in relation to the sample, in this case corresponding to Study 2. In view of the possible reluctance of certain workers to participate in the study, after consulting with the HR departments of different companies, it was decided to collect the lowest quantity of sociodemographic data so that no worker could be identified. Thus, the age of the participants was not asked. 

Finally, the complex emotional situation resulting from the pandemic should be taken into account. In general, individuals have experienced unstable feelings and these may have changed their perception, influencing the responses to the questionnaires [42]. Indeed, it should be noted that this research uses self-report measures that may include cognitive bias [43]. Despite possible limitations, this research provides support for the theoretical basis and offers interesting insights into the relationship between the COVID-19 pandemic as traumatic event, mental health, and fear of COVID-19.

### 4.2. Implications

Despite these limitations, some practical and theoretical implications can be drawn from the present study. Starting from the latter, previous research has highlighted a significant gap in the current literature on PTSD (e.g., [44]) in particular regarding the absence of recent studies related to the Horowitz model and cognitive appraisal theories. Consequently, one of the aims of this study was to fill this gap. We found support for the role of intrusive thoughts as the primary factor in the onset of post-traumatic symptoms, which may precede hyperarousal and avoidance responses. Moreover, our results suggest that hyperarousal, compared to avoidance, plays a mediating role in the relationship between intrusion and individual outcomes. Future research should further investigate these relationships, analyzing the impact of hyperarousal net of intrusion. Hence, our findings shed new light on the Horowitz’s theoretical approach and CATS theory to understanding post trauma reactions and explaining the effects of mass traumatic events as the COVID-19 pandemic. 

From a practical point of view, the results of this study provide relevant information for clinical and prevention programs, demonstrating the importance of considering intrusive thoughts and hyperarousal as primary symptoms to be treated within the COVID-19 pandemic. Since PTSD is generally associated with poor functioning and a low quality of life (e.g., [45]), our results show that this could also happen within the COVID-19 experience, with detrimental consequences for psychological well-being. Based on our findings, further studies need to be designed to investigate the efficacy of specific interventions for PTSD. These interventions, even within organizational contexts, should support individuals towards a better understanding of their inner feelings and inner self through empowerment processes [46]. The implementation of workplace health promotion programs is advisable, as practical interventions have demonstrated a positive return on investment [47,48]. Furthermore, emotional skills programs could have a significant impact on individual and work outcomes [49,50]. Healthcare professionals, such as psychologists, should provide programs aimed at both the acquisition of healthy life skills and psychoeducation. On the one hand, methods of positive interactions with oneself and others should be proposed. On the other hand, useful information for self-screening should be provided, including information about the negative consequences of stress related to COVID-19 and PTSD.

## 5. Conclusions

The COVID-19 pandemic has disrupted the lives of individuals across the globe on a personal, social, and occupational level, with negative consequences for psychological functioning. This study brought new insight on the relationship between PTSD and individual outcomes, such as fear of COVID-19 and mental health, during the COVID-19 pandemic in Italy. Furthermore, to the best of our knowledge, this is the first research that investigates the relationship between intrusion as a central dimension of PTSD and two different types of individual outcomes using a mediation model. More specifically, the hypotheses are supported because intrusive thoughts had a significant negative effect on hyperarousal, compared with avoidance, and the former partially mediated the effect of intrusion on fear of COVID-19 and mental health. The present study analyzes hyperarousal and avoidance, defined according to the Horowitz model and CATS theory, in order to provide a new explanation for PTSD and its consequences. As the results are limited by the Italian context and the cross-sectional design, further studies using longitudinal designs and considering other possible mediators and cultural contexts are needed to support the conclusions. Nevertheless, these findings provide interesting theoretical and practical insights.

## Figures and Tables

**Table 1:** Socio-demographic characteristics of the sample (Study 1, *N* = 627).

Characteristics	
Age (Mean, SD)	31.4, 13.9
Gender	(%)
Male	35.2
Female	64.8
Marital status	(%)
Single	71.5
Married	28.5
Educational level	(%)
Primary	1.9
Secondary	48.6
University	39.4
Master	8.3
PhD	1.8
Job status	(%)
Student	22.3
Unemployed	17.6
Employed	60.1

**Table 2:** Socio-demographic characteristics of the sample (Study 2, *N* = 495).

Characteristics	
Gender	(%)
Men	67.7
Women	32.3
Organizational seniority	(%)
4 years or less	19.1
5–9 years	43
10–20 years	21.8
More than 20 years	16.1
Job category	(%)
Chief	3.1
Middle management	20.1
Employee	76.8

**Table 3:** Items and psychometric properties of the fear of COVID-19 questionnaire.

Item	Factorloading *	Item-Total Correlation	Mean (SD)	Skewness	Kurtosis
1. I am afraid of contracting the virus	0.477	0.69	3.61 (1.09)	−0.419	−0.475
2. I am afraid of the possibility of buying potentially contaminated goods	0.488	0.70	2.71 (1.28)	0.234	−0.995
3. I am afraid of buying goods in shops	0.567	0.75	2.44 (1.15)	0.405	−0.683
4. I am afraid of using urban transport	0.691	0.83	4 (1.15)	−1.087	0.425
5. I am afraid of using trains	0.763	0.87	3.72 (1.23)	−0.728	−0.410
6. I am afraid of using planes	0.651	0.81	3.61 (1.24)	−0.558	−0.711
7. I am afraid of going to facilities to use health services (general practitioner, hospitals, etc.)	0.502	0.71	3.58 (1.15)	−0.595	−0.034
8. I am afraid of going to facilities for basic necessities (bank, post office, supermarket, etc.).	0.642	0.80	2.96 (1.06)	−0.082	−0.555

Note: * Extraction method: Factor loadings using the extraction method: unweighted least squares. SD = standard deviation.

**Table 4:** Mean, standard deviation, and correlations among the study variables in Study 1.

	M	SD	1	2	3
1. Intrusion	3.40	1.07			
2. Hyperarousal	2.75	1.08	0.65 **		
3. Avoidance	2.84	1.19	0.53 **	0.75 **	
4. Fear of COVID-19	26.63	7.21	0.42 **	0.41 **	0.36 **

Note: *N* = 627. M = mean. SD = standard deviation. ** *p* < 0.01.

**Table 5:** Mean, standard deviation, and correlations among the study variables in Study 2.

	M	SD	1	2	3
1. Intrusion	2.92	1.08			
2. Hyperarousal	2.09	0.96	0.63 **		
3. Avoidance	2.12	1.11	0.52 **	0.70 *	
4. Mental health	1.98	0.45	−0.25 **	−0.49 **	−0.43 **

Note: *N* = 495. M = mean. SD = standard deviation. * *p* < 0.05; ** *p* < 0.01.

## Data Availability

The data presented in this study are available on request from the corresponding author. The data are not publicly available due to privacy issues.
